# *Lacticaseibacillus paracasei sh2020* induced antitumor immunity and synergized with anti-programmed cell death 1 to reduce tumor burden in mice

**DOI:** 10.1080/19490976.2022.2046246

**Published:** 2022-03-08

**Authors:** Shi-Long Zhang, Bing Han, Yu-Qin Mao, Zheng-Yan Zhang, Zhan-Ming Li, Chao-Yue Kong, You Wu, Guo-Qiang Chen, Li-Shun Wang

**Affiliations:** a Center for Traditional Chinese Medicine and Gut Microbiota, Minhang Hospital, Fudan University, Shanghai, China; b Institute of Fudan-Minhang Academic Health System, Minhang Hospital, Fudan University, Shanghai, China; c State Key Laboratory of Oncogenes and Related Genes, and Chinese Academy of Medical Sciences Research Unit (NO.2019RU043), Renji Hospital, Shanghai Jiaotong University School of Medicine, Shanghai, China

**Keywords:** Colorectal cancer, immunotherapy, anti-PD-1, gut microbiota, probiotics, tumor microenvironment

## Abstract

The gut microbiota was emerging as critical regulatory elements in shaping the outcome of cancer immunotherapy. However, the underlying mechanisms by which the gut commensal species enhance antitumor immunity remain largely unexplored. Here, we show that the gut microbiota from healthy individuals conferred considerable sensitivity to anti-PD-1 in the colorectal cancer (CRC) tumor-bearing mice, whereas gut microbiota from CRC patients failed to do so. By 16S rRNA gene sequencing, we identified *Lactobacillus* that was significantly increased in the mice with good response to anti-PD-1, and significantly correlated with anti-tumor immunity. After a series of screening, we isolated a novel *Lacticaseibacillus* strain, named *L. paracasei sh2020. L. paracasei sh2020* showed the most notable anti-tumor immunity in the mice with gut dysbiosis. Mechanistically, the antitumor immune response elicited by *L. paracasei sh2020* was dependent on CD8^+^ T cell. *In vitro* and *in vivo* studies revealed that *L. paracasei sh2020* stimulation triggered the upregulated expression of CXCL10 in the tumors and subsequently enhanced CD8^+^ T cell recruitment. Meanwhile, the modulation of gut microbiota caused by *L. paracasei sh2020* enhanced its antitumor effect and gut barrier function. Overall, our study offered novel insights into the mechanism by which gut microbiota shaped the outcome of cancer immunotherapy and, more importantly, the novel strain *L. paracasei sh2020* might serve as an easy and effective way to promote anti-PD-1 effect in clinical practice.

## Introduction

Colorectal cancer (CRC) remains one of the common digestive tract tumors with increasing incidence and mortality worldwide. The prognosis of CRC remains dismal despite improved diagnostic and treatment strategies. In recent years, immunotherapy has become an emerging way in the treatment of solid tumors,^1^ which harness the immune system to produce anti-tumor effects. Anti-programmed cell death 1 (anti-PD-1) immunotherapy, as novel immunotherapy drug, has achieved favorable results in several advanced cancers.^2,3^ In contrast to other tumor types, CRC patients who benefit from anti-PD-1 are dismally limited.^4,5^ Therefore, how to make more CRC patients benefit from immunotherapy remains a hot topic in this field.

Recently, gut microbiota has fueled great enthusiasm in immunotherapy. Our recent publication has demonstrated that targeting gut microbiota using dietary fiber facilitated the anti-PD-1 efficacy in the gut humanized avatar mouse models.^6^ Moreover, the dietary fiber shifts the gut microbial community toward enrichment in *Akkermansia, Lactobacillus* and *Bifidobacterium*, which were significantly associated with good response to anti-PD-1 therapy.^7–9^ More recently, Mager L.F. *et al* isolated three bacterial species, including *B. pseudolongum, L. johnsonii* and *Olsenella*, and found that oral administration of these species could significantly enhance the therapeutic effect of immune checkpoint inhibitors in tumor models.^10^

Normal gut microbiota is a huge but relatively unexplored treasure trove.^11^ Although some specific bacteria have been associated with increased anti-tumor immunity, the research about single gut bacteria in immunotherapy is still far from experimentally clarified.^12^ Furthermore, the exact molecular mechanisms through which these gut microbes swayed immunotherapy has rarely been studied.^13^ In this study, we found that *Lactobacillus* was significantly associated with good response to anti-PD-1 in the tumor models of CRC. After a series of screening, we isolated and identified a novel *Lactobacillus* strain, named *L. paracasei sh2020*, which showed the most notable anti-tumor immunity in the gut humanized avatar mouse models. Subsequently, we put more efforts on the underlying mechanisms of the antitumor immunity induced by *L. paracasei sh2020*. Additionally, the favorable effects of *L. paracasei sh2020* on gut homeostasis were confirmed in the context of anti-PD-1 therapy.

## Results

### Gut microbiota from healthy donors but not from CRC patients improved the efficacy of anti‑PD-1 in recipient mice

**Figure 1.**
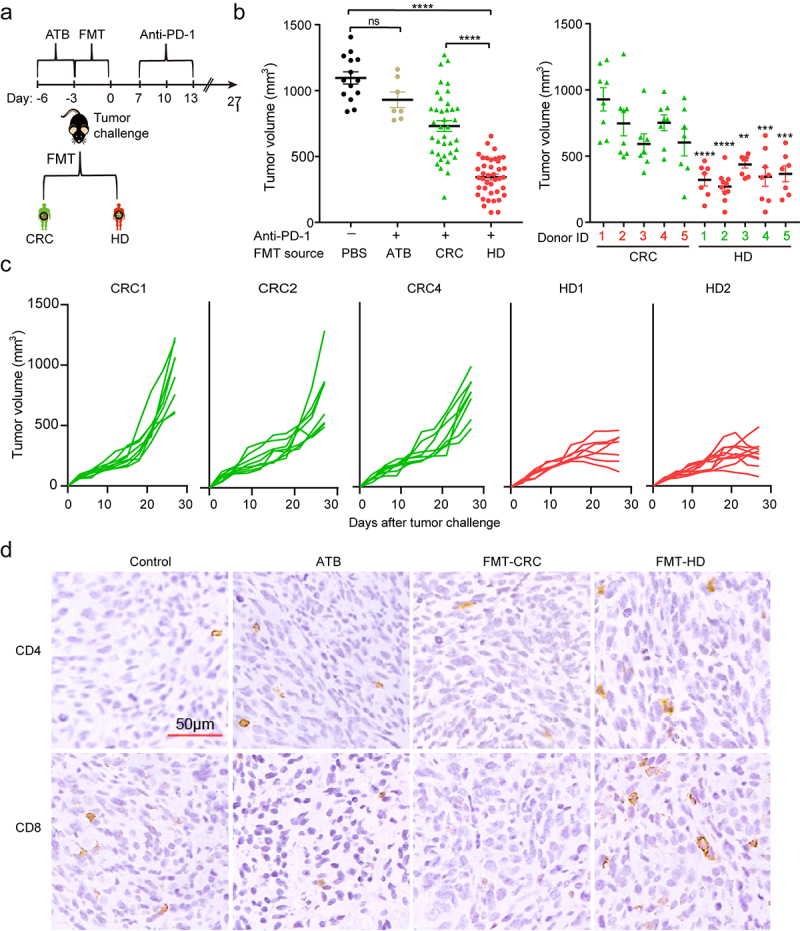
The gut microbiota from healthy donors, but not from CRC patients, improved therapeutic efficacy of anti‑PD‑1. (a) Experimental design: fecal microbiota transplantation (FMT) was performed after 3 days of ATB administration in mice. Five days later, MC38 cells were inoculated subcutaneously and anti-PD-1 was intraperitoneally administered every 3 days starting on day 7, in total three times. (b) The tumor volume of pooled groups of mice (left) and individual groups of mice (right) receiving gut microbiota from the CRC patients (CRC1-5) or healthy individuals (HD1-5) (n = 8–10). **P < .01, ***P < .001, ****P < .001 versus control (pooled data). (c) Individual tumor growth curves of the mice receiving gut microbiota from CRC patients (CRC1, CRC2, and CRC4) or healthy individuals (HD1, HD2) during treatment with anti-PD-1. (d) Representative images of IHC staining of CD4, and CD8 for tumors from each group.

Five healthy donors (HD) and newly diagnosed CRC patients were collected from our institute. The detailed characteristics of all participants were listed in **Table S1**. Compared to HD, the CRC patients were characterized by decreased α-diversity (**Figure S1a-b**), distinct β-diversity (**Figure S1c**) as well as disturbed gut microbiota profiles (**Figure S1d-f**) consistent with recent studies.^14,15^ To determine whether and if so, to what extent, gut microbiota has influenced the response to anti-PD-1, we took advantage of avatar mouse models, which were gavaged with a broad-spectrum antibiotic cocktail (ATB) to depleted endogenous microbiota and subsequently humanized with feces from HD or CRC donors using fecal microbial transplantation (FMT). Such “humanized” mice were inoculated with syngeneic MC38 cells as described in our recent publication to investigate the role of gut microbiota in anti-PD-1 therapy (Figure 1a).^6^ As shown in Figure 1b, anti-PD-1 differently influenced tumor progression in an individual group of mice. Tumor growth was considerably inhibited in mouse recipients of healthy microbiota compared to mouse recipients of CRC microbiota. Specifically, in an individual group of mice, three individual groups (CRC1, CRC2, and CRC4) resulted in faster tumor growth, showing an impaired efficiency of anti-PD-1, while in the mice colonized with microbiota from HD, two individuals (HD1 and HD2) recipient mice almost completely abrogated the tumor progression (Figure 1c). Furthermore, increased CD4^+^ cells and CD8^+^ cells, and decreased Foxp3^+^ cells were observed in the tumors of the HD group than that of the CRC group, thereby fostering “hot” tumors (Figure 1d).

Collectively, gut microbiota from CRC tended to suppress the efficacy of anti-PD-1 treatment, which might partly explain the low response to anti-PD-1 among CRC patients. On the other hand, gut microbiota from HD conferred sensitivity to anti-PD-1, providing the possibility that there existed effector bacteria in the gut, which might play a dominant role in facilitating anti-PD-1 therapy.

### Distinct gut microbiota development in recipient mice colonized with fecal microbiota from CRC and healthy donors

**Figure 2.**
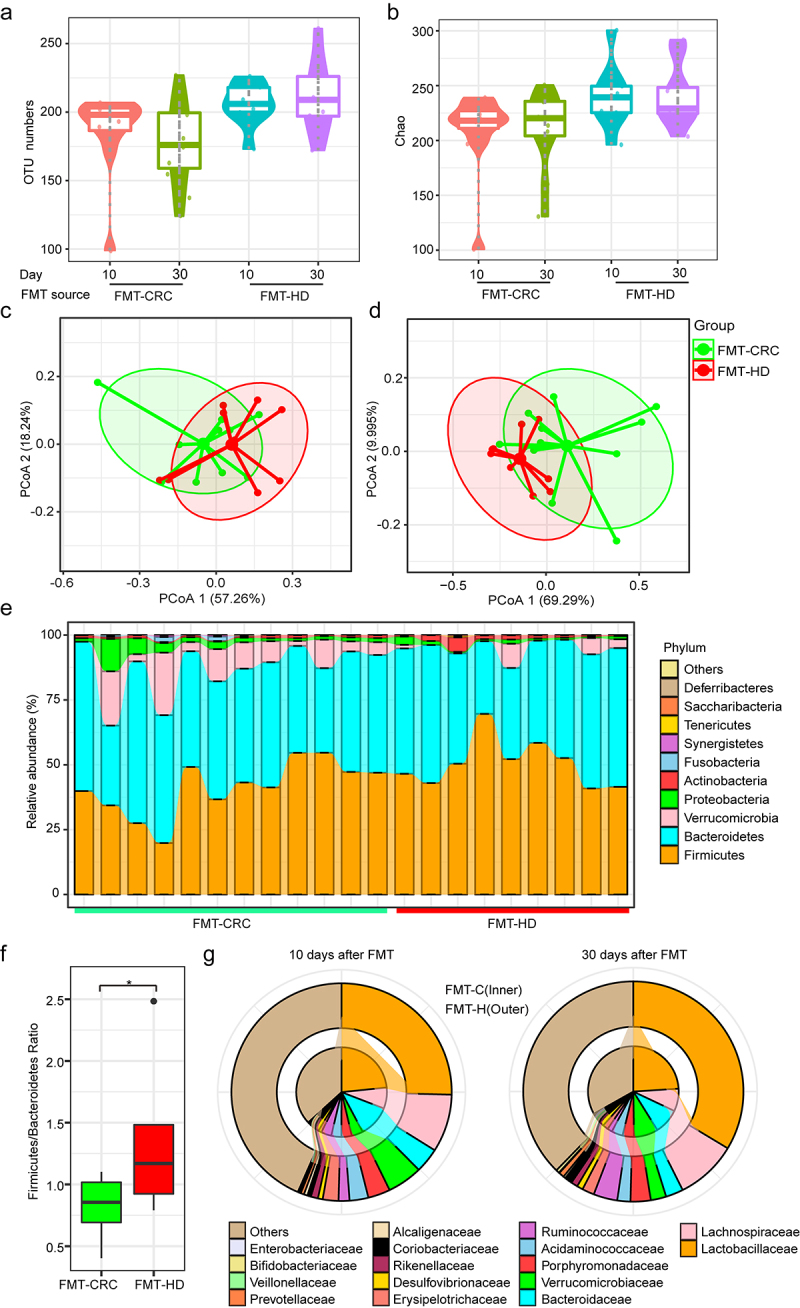
The gut microbiota from healthy donors restored normobiotic ecology of gut microbiota. (a-b) Changes in the α-diversity between the FMT-CRC and FMT-HD group was determined using the OUT numbers (a), and Chao index (b) (n = 7). (c-d) PCoA of β-diversity using the Bray-Curtis dissimilarity metric among samples of individual groups of mice in each group at day 10 (c), and day 30 (d). (e) Component proportion of bacterial phylum in each group. (f) Firmicutes-to-Bacteroidetes ratio in individual groups of mice in each group at days 30. (g) Comparison of relative abundance of bacterial class level between FMT-CRC (inner rings) and FMT-HD group (outer ring) at days 10 (left), and 30 (right). *P < .05.

We conducted 16S rRNA sequencing to analyze the fecal samples collected from mice at day 10 and 30, respectively. The number of observed OTUs was significantly higher in the FMT-HD group at days 30 (Figure 2a-b), which reflected a higher community richness of FMT-HD. Upon analyzing β-diversity across groups, the overall community at day 10 revealed a distinct clustering pattern between the two groups (Figure 2c) and was sustained at 30 days (Figure 2d). We then assessed the landscape of gut microbiota overtime in two groups. No significant change was observed among the most abundant phyla, including *Firmicutes* and *Bacteroidetes* (Figure 2e). However, the *Firmicutes/Bacteroidetes* ratio in FMT-HD group was significantly higher than that in the FMT-CRC group, suggesting a better capacity to maintain normal gut homeostasis (Figure 2f). ^16,17^ At the lower taxonomic level, FMT-HD group showed restoration toward gut normobiosis. For instance, several well-known commensals used in probiotic preparations,^18,19^ including *Lactobacillaceae, Bifidobacteriaceae, Erysipelotrichaceae*, and *Ruminococcaceae*, which are immunomodulatory SCFA-producing taxa, and associated with healthy gut ecosystems,^20^ increased upon FMT from healthy individuals at day 10. At day 30, the relative abundance of *Lactobacillaceae, Bifidobacteriaceae*, and *Ruminococcaceae* increased progressively in the FMT-HD group (Figure 2g). These findings demonstrated the distinct gut microbiota development between the recipient mice colonized with fecal from CRC and HD.

### Gut microbial signature potentially associated with anti-PD-1 efficacy

**Figure 3.**
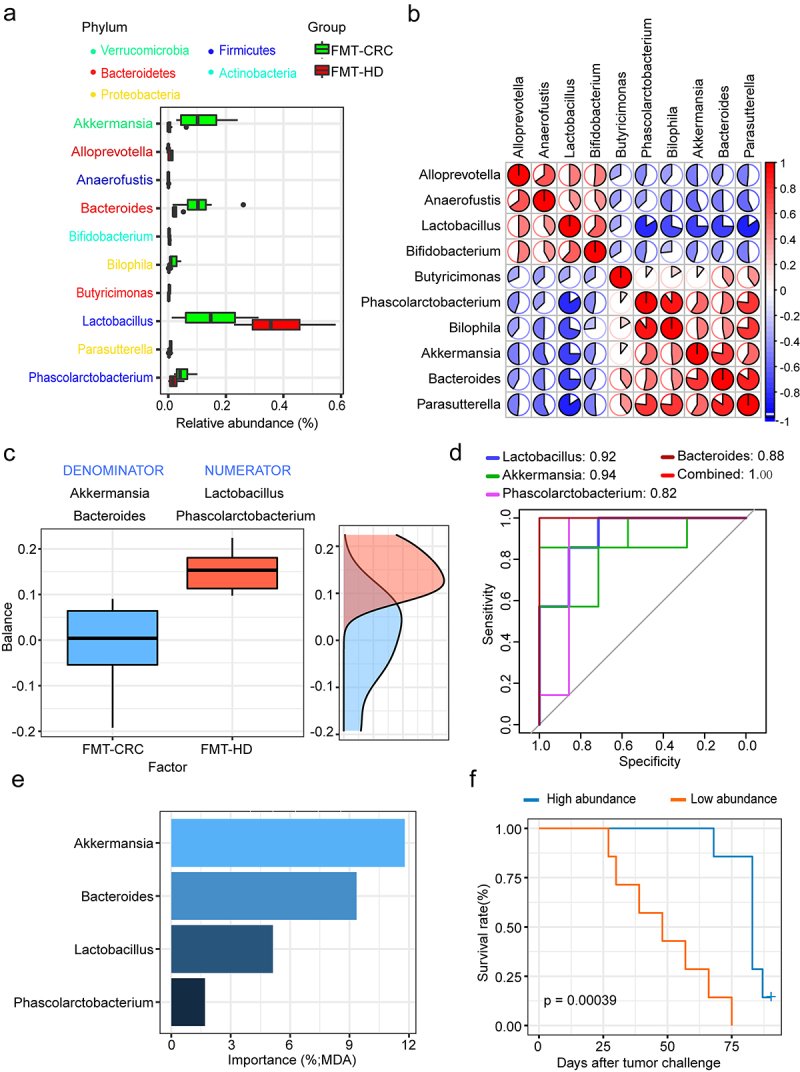
*Lactobacillus* was positively associated with antitumor immune response. (a) Differential relative abundance of taxa at the genus level between the FMT-CRC and FMT-HD group (n = 7). (b) Relationship of genera with different abundances. Lactobacillus was negatively correlated with almost all other genera with different relative abundances. (c) Gut microbial signature associated with anti-PD-1 outcomes in mice. (d) ROC analysis of the power of four genera as predictive of anti-PD-1 outcomes. (e) The ranking of four genera by their importance to the gut microbial community. (f) Kaplan-Meier survival analysis based on the abundance of *Lactobacillus* in the tumor-bearing mice (n = 14). MDA, mean decrease in accuracy.

To identify the microbial taxa that might be potentially associated with the improved efficacy of anti-PD-1, we performed a differential taxa analysis between the two groups. In total, 10 taxa were differentially abundant at the genus level. Notably, FMT-HD group consistently exhibited increased relative abundance of *Lactobacillus* (Figure 3a). Furthermore, *Lactobacillus* was negatively correlated with almost all other genera, with different relative abundances between the two groups (Figure 3b). A microbial signature with high accuracy in predicting the outcomes of anti-PD-1 therapy was identified using *selbal*.^21^ This signature consists of the two genera, *Lactobacillus* and *Phascolarctobacterium* as a numerator, and *Akkermansia* and *Bacteroides* as a denominator (Figure 3c). More importantly, the signature produced an area under the receiver operating characteristic curve (AUC) of 1.00 (Figure 3d). A positive balance value in the FMT-HD group suggested that the two genera in the numerator had a much higher abundance than that in the denominator (*Akkermansia* and *Bacteroides*). *Lactobacillus*, the abundance of which increased most in the FMT-HD group, were also ranking as the top three most important taxon to the microbiota community (Figure 3e).

We then stratified all gut humanized mice based on the median relative abundance of *Lactobacillus*. Kaplan–Meier analysis showed significantly longer survival that was observed in the *Lactobacillus* high group (Figure 3f). Indeed, recent studies demonstrate that some strains of *Lactobacillus* can modulate the immune response against tumors in murine model.^22,23^ Collectively, we thus hypothesized that members of this genus might represent a major component underlying the improved antitumor immune effects in FMT-HD group.

### Lactobacillus *cocktail improved response to anti-PD-1 in tumor-bearing mice*

**Figure 4.**
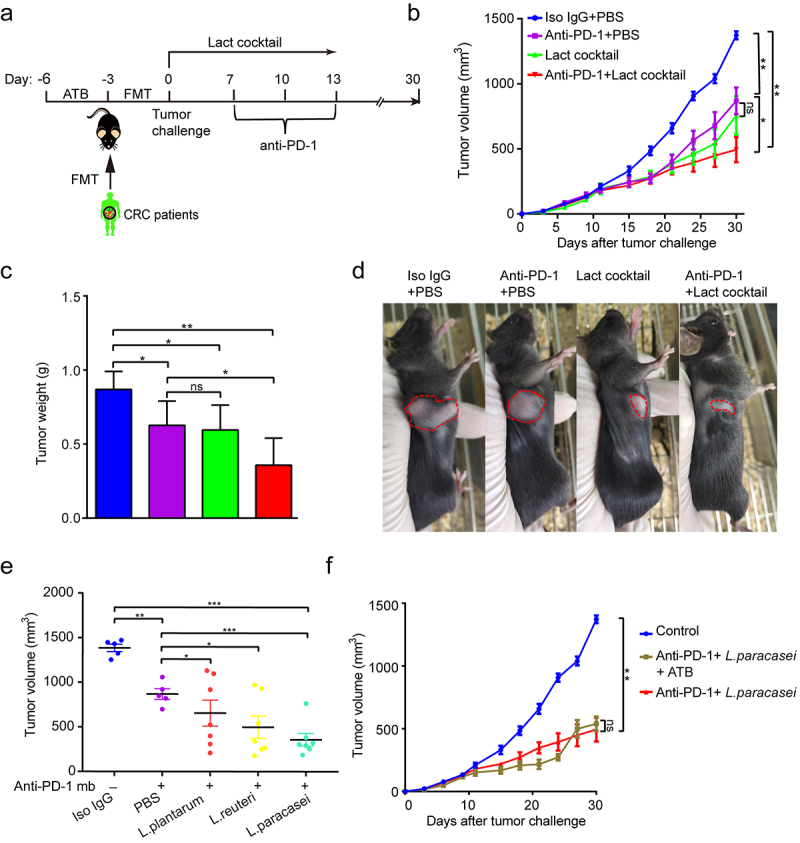
*Lactobacillus* cocktail improved response to anti-PD-1 in the mice with gut dysbiosis. (a) Experimental design: FMT from C1 patient was conducted after 3 days of ATB treatment in SPF mice. After the FMT complement, the tumor-bearing mice were orally administrated with Lactobacillus cocktail once a day for 10 days after tumor challenge. (b) Tumor growth from the mice after treatment with isotype IgG, or anti-PD-1 alone or *Lactobacillus* cocktail alone, anti-PD-1 combined with *Lactobacillus* cocktail (n = 6–7). (c) Tumor weight of the tumor-bearing mice in each group (n = 6–7). (d) Representative photograph of tumors on day 20 after tumor challenge. (e) Tumor volumes from the tumor-bearing mice receiving different strains. (f) Tumor growth in the tumor-bearing mice pre-treated with antibiotic cocktail (n = 6–7). ns, no significant difference, *P < .05, **P < .01, ***P < .001.

To determine causality for at least one *Lactobacillus* member, all stools of the healthy donors were cultured with Man Rogosa Sharpe medium, which is a selective cultural medium designed to favor the *Lactobacillus* growth. As a result, three bacterial species, including *L. reuteri, L. plantarum*, and *L. paracasei*, were able to be cultured and identified by 16S rRNA. We thus created a live cocktail with the three species and administered it via oral gavage to gut humanized mice using the fecal from CRC1 donor (Figure 4a). Notably, this cocktail significantly reversed the impaired efficacy of anti-PD-1 observed in the recipient mice (Figure 4b-d).

Among this cocktail, we further mined the functional strain that improved the efficacy of anti-PD-1. As shown in Figure 4e, the isolated *L. plantarum* had no apparent effect on the tumor growth when combined with anti-PD-1. The isolated *L. reuteri* showed mild anti-tumor effects. In contrast, the isolated *L. paracasei* elicited the most notable anti-tumor ability, demonstrating that *L. paracasei* was likely the key functional strain responsible for the enhanced anti-tumor immunity. Interestingly, the isolated *L. paracasei* retained its favorable effect in controlling the tumor growth in the mice basically depleted of gut microbiota (Figure 4f). In addition, neither morphological nor pathological damage was found in major organs **(Figures S2**), indicating that the isolated *L. paracasei* did not exert a significant adverse influence in normal tissues. Then, the whole genome of the isolated *L. paracasei* was sequenced and assembled using Oxford Nanopore combined with Illumina high throughput next-generation sequencing technologies.^24^ The genome comparative analyses among the isolated *L. paracasei* and other 26 *L.casei* strains available in the NCBI database were presented in **Figures S3** and Table S2, suggesting that the isolated *L. paracasei* was a novel strain, and thus we named it *L. paracasei sh2020*.

### Transcriptome analysis revealed upregulation of T cell trafficking-associated genes within tumors following L. paracasei sh2020

**Figure 5.**
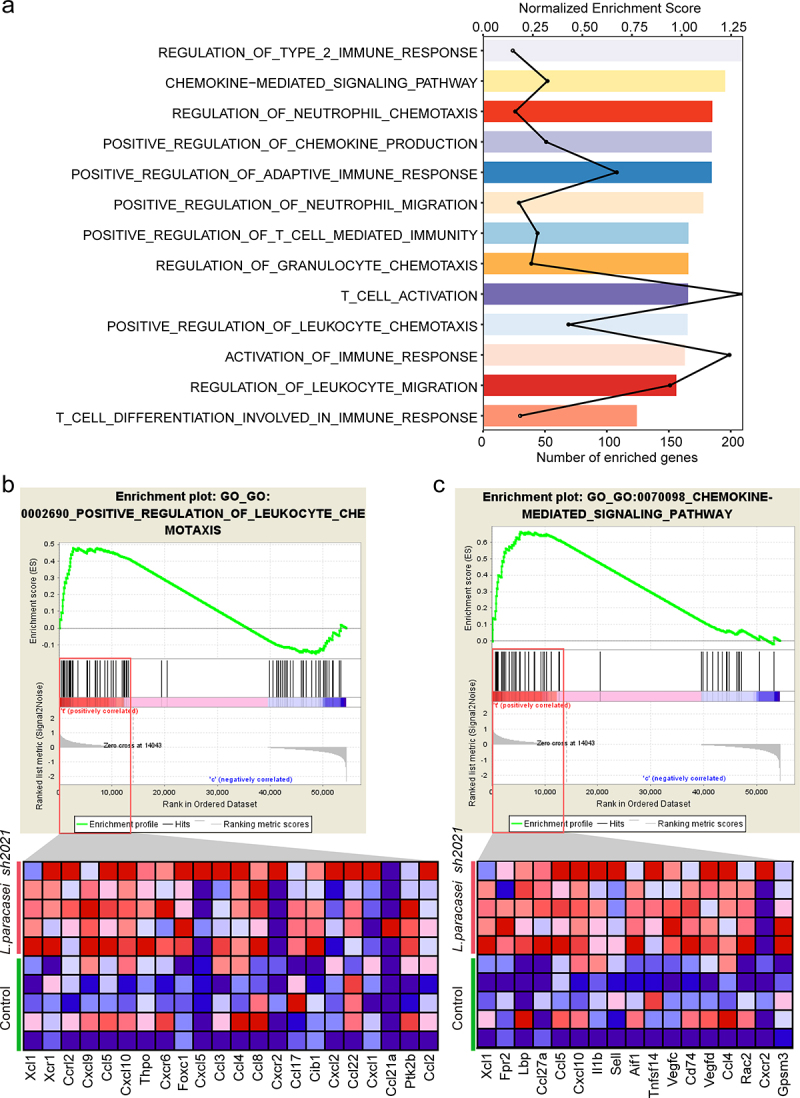
Transcriptome analysis revealed the increased T cell trafficking-associated genes following *L. paracasei sh2020*. (a) Enriched gene sets in *L. paracasei sh2020*-treated tumors identified by GSEA (n = 5). (b-c) GSEA enrichment plots of positive regulation of leukocyte chemotaxis and chemokine-mediated signaling pathway that were enriched in the *L. paracasei sh2020*-treated tumors were shown (n = 5).

To explore the potential mechanisms of *L. paracasei sh2020*, we performed RNA-seq analysis of tumor tissues harvested from the mice treated with vehicle, or *L. paracasei sh2020*. First, GSEA analysis identified high-level categories, such as regulation of T cell mediated immunity, leukocyte migration, positive regulation of adaptive immune response, were enriched in the *L. paracasei sh2020-*treated tumors (Figure 5a). Notably, this group promoted the expression of T cell chemoattractant chemokines (Figure 5b-c), such as CXCL9, CXCL10, and CXCL11, previously described to be involved in modulating the tumor immune environment.^25^ Next, the RNA-seq data were then analyzed to estimate the tumor-infiltrating immune cell. We also found that there was a greater proportion of CD8^+^ T cells in the *L. paracasei sh2020*-treated tumors (**Figure S4**). These results suggested that *L. paracasei sh2020* might elicit chemokines expression in the tumors, which ultimately promoted CD8^+^ T cell infiltration into the tumor beds.

### *CD8^+^ T cell mediated the anti-tumor immunity of* L. paracasei sh2020

**Figure 6.**
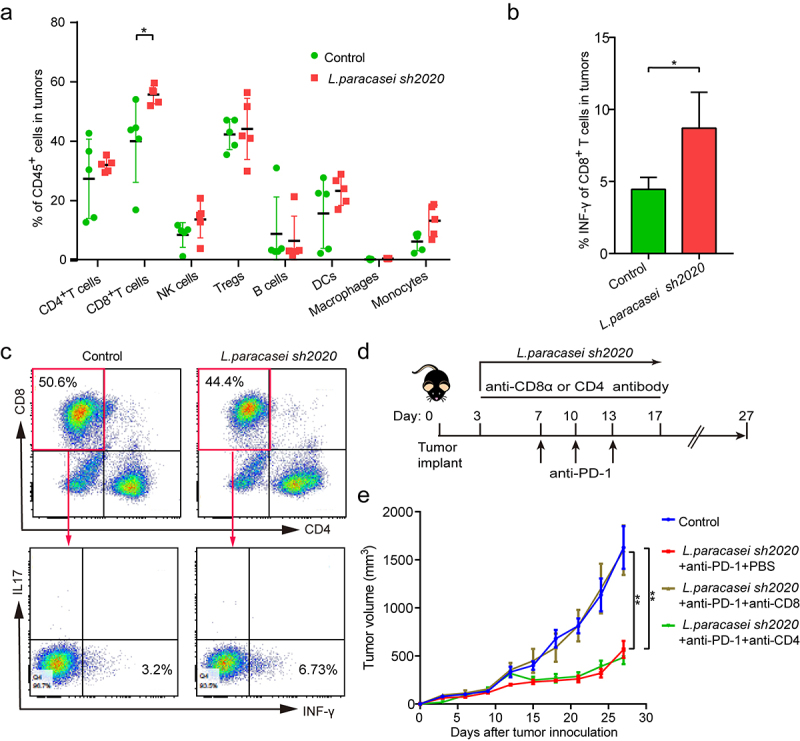
CD8^+^ T cells were required for anti-tumor effect of *L. paracasei sh2020*. (a) The percentage of tumor-infiltrating immune cell in individual mice in the control and *L. paracasei sh2020*-treated group (n = 5). (b-c) The expression of INF-γ on the CD8^+^ T cells within tumors from mice in the control and *L. paracasei sh2020*-treated group at the end of the experiment, representative flow sample and statistics were showed in c and d, respectively (n = 4–5). (d) Experimental design: C57BL/6 mice were implanted subcutaneously with 5.0 × 10^5^ MC38 cells and treated with anti-PD-1 + *L. paracasei sh2020*. The anti-mouse CD8α or CD4 antibody was initiated 1 day before anti-PD-1 + *L. paracasei sh2020* treatment and continued twice a week for two weeks. (e) Tumor growth in the tumor-bearing mice treated with *L. paracasei sh2020* and depleted for CD4^+^ or CD8^+^ T cells (n = 5–6). *P < .05, **P < .01.

Next, flow cytometry was used to examine the immune cell infiltrates of control and *L. paracasei sh2020*-treated tumors, which showed a prominent expansion of CD8^+^ T cells in *L. paracasei sh2020*-treated tumors, and no significant changes were found in other immune subsets (Figure 6a). Furthermore, IFN-γ^+^CD8^+^ T cells were significantly increased after *L. paracasei sh2020* administration (Figure 6b-c). Moreover, these CD8^+^T cells had improved activation status following *L. paracasei sh2020*, since ICOS was upregulated on them **(Figures S5)**. To identify which T cell subsets were required for the efficacy of *L. paracasei sh2020*, the CD4^+^ or CD8^+^ T cells were depleted using *in vivo* neutralizing antibodies against CD8, CD4 (Figure 6d). ^26^ Depletion of CD8^+^ T cells completely abrogated the effect of *L. paracasei sh2020*, suggesting that CD8^+^ T cells were indispensable for the anti-tumor immunity of *L. paracasei sh2020*. In contrast, depletion of CD4^+^ T cells did not influence the anti-tumor effect of *L. paracasei sh2020* (Figure 6e). Collectively, *L. paracasei sh2020* promoted CD8^+^ T cell infiltration and induced a CD8^+^ T cell-dependent anti-tumor immunity.

### L. paracasei sh2020 *increased the expression and release of CXCL10 in the tumors*

**Figure 7.**
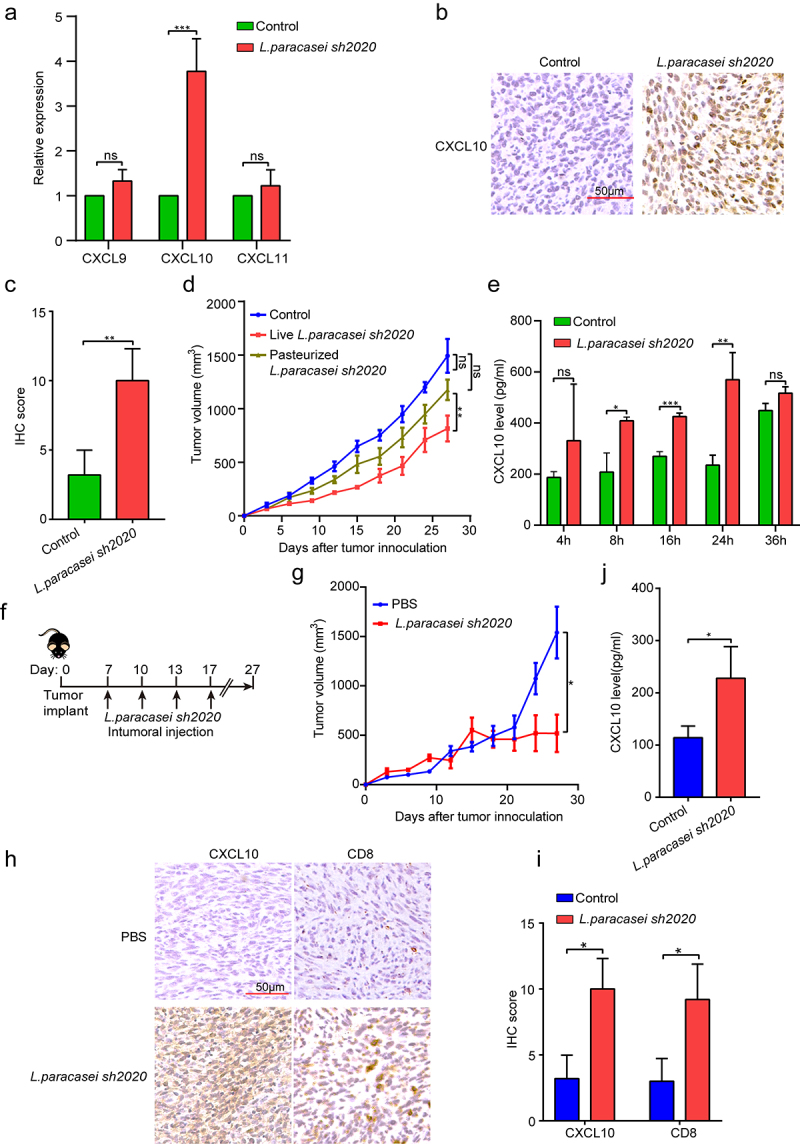
*L. paracasei sh2020* promoted the expression and secretion of CXCL10 *in vivo* and *in vitro*. (a) The expression of CXCL9, CXCL10 and CXCL11 in tumor tissues from control and *L. paracasei sh2020-*treated mice was detected by qRT-PCR. (b-c) Representative images (b) and quantification (c) of IHC staining of CXCL10 in the tumor tissues from control and *L. paracasei sh2020*-treated tumors. (d) Tumor growth in each group. (e) The levels of CXCL10 in the conditioned medium. (f-g) Tumor growth in the tumor-bearing mice with intratumoral injection of *L. paracasei sh2020* (n = 5–6). (h-i) Representative images (h) and quantification (i) of IHC staining of CXCL10 and CD8 in each group (n = 4–5). (j) The serum levels of CXCL10 were examined by ELISA. ns, no significant difference, *P < .05, **P < .01, ***P < .001.

Our next goal was to explore the underlying mechanisms by which *L. paracasei sh2020* facilitate the CD8^+^ T cell infiltration in the tumors. Analysis of RNA-seq data showed that specific chemokines that promoted T cell recruitment were upregulated after *L. paracasei sh2020* administration, suggesting the pivotal role of *L. paracasei sh2020* in boosting chemokine expression and release in tumor microenvironment. Among them, CXCL10 showed the most increase compared with other chemokines (Figure 5b-c). qRT-PCR showed that *L. paracasei sh2020* made a drastic increase in the level of CXCL10 in tumor tissue, whereas no changes were found in CXCL9, and CXCL11 (Figure 7a). This observation was also confirmed by IHC of CXCL10 in the tumors (Figure 7b-c).

Then, *L. paracasei sh2020* was pasteurized and the dead *L. paracasei sh2020* abrogated the tumor control observed in the tumor-bearing mice (Figure 7d), indicating the live bacteria but not the bacterial debris, induced antitumor immunity. Hence, live *L. paracasei sh2020* was cultured and used to investigate its role in the expression and release of CXCL10 *in vitro*. The tumor cells were incubated with *L. paracasei sh2020* for 4 h, 8 h, 16 h, 24 h, and 36 h, respectively, secretion of CXCL10 protein was measured using ELISA assays. *L. paracasei sh2020* promoted CXCL10 secretion after 8 h, 16 h, 24 h, and 36 h, and highest level of CXCL10 protein was achieved after 24 h of incubation (Figure 7e). To further confirm this result *in vivo*, intratumoral injection of live bacteria was performed according to the recent studies.^27,28^ We found that intratumoral injection of live *L. paracasei sh2020* also considerably prevented tumor growth (Figure 7f-g), induced CXCL10 expression, and CD8^+^ T cell infiltration in the tumor beds (Figure 7h-i). In addition, increased CXCL10 levels were observed in the serum of these mice compared to those of the controls (Figure 7j). Together, *L. paracasei sh2020* promoted CXCL10 secretion *in vitro* and *in vivo*, which was known to facilitate recruitment of cytotoxic T cells into tumors.^29,30^

### *CXCL10 controlled CD8^+^ T cell infiltration mediated by* L. paracasei sh2020

**Figure 8.**
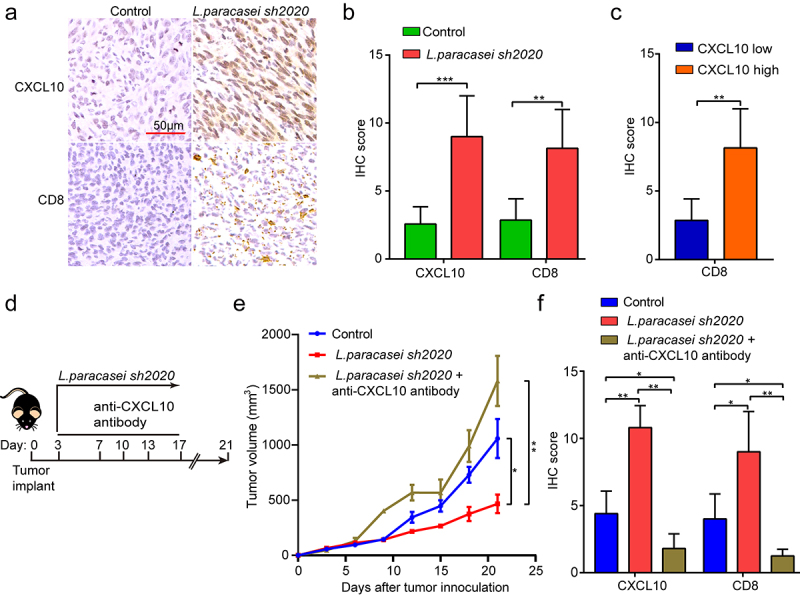
CXCL10 controlled CD8^+^ T cell migration and the effect of *L. paracasei sh2020 in vivo*. (a-b) Representative images of IHC staining of CD8 and CXCL10 (a), and quantification (b) for the control and *L. paracasei sh2020*-treated tumors (n = 6–7). (c) IHC analysis of CD8 in tumors, which were divided into two groups according to CXCL10 high and low expression. (d) Experimental design: C57BL/6 mice were implanted subcutaneously with 5.0 × 10^5^ MC38 cells and was treated with control vehicle or anti-CXCL10 antibody by intraperitoneal injection, every 3 days starting on D3, in total three times. The mice were given *L. paracasei sh2020* with a dose of 1.0 × 10^9^ CFU by gavage starting from D0 to D13. (e) Tumor growth in tumor-bearing mice in d. (f) Quantification of IHC staining of CXCL10 and CD8 in the tumors after neutralizing CXCL10 *in vivo* (n = 4–5). ns, no significant difference, *P < .05, **P < .01, ***P < .001.

Furthermore, the CXCL10 and CD8 expressions were detected in the control and *L. paracasei sh2020*-treated tumors using IHC (Figure 8a). It was found that *L. paracasei sh2020*-treated tumors showed an increase in CD8 expression, as well as CXCL10 staining. In contrast, control tumors with a low score of CXCL10 showed lower levels of CD8 stain (Figure 8b). Collectively, a negative correlation between CXCL10 and CD8^+^ T cell infiltration was observed in the tumor tissues (Figure 8c). To further investigate the role of CXCL10 on the effect of *L. paracasei sh2020 in vivo*, we blocked CXCL10 using anti-CXCL10-neutralizing antibody in the *L. paracasei sh2020*-treated mice, showing that neutralizing CXCL10 *in vivo* completely dampened the effect of *L. paracasei sh2020* (Figure 8d-e). This rescue of the tumor growth was associated with significantly decreased CD8^+^ T cell infiltration (Figure 8f**, and Figure S6)**, suggesting that CXCL10-mediated CD8^+^ T cell infiltration played a crucial role in *L. paracasei sh2020*-induced tumor inhibition.

### *Oral intake of* L. paracasei sh2020 *maintained gut microbiota homeostasis in the context of anti-PD-1 therapy*

**Figure 9.**
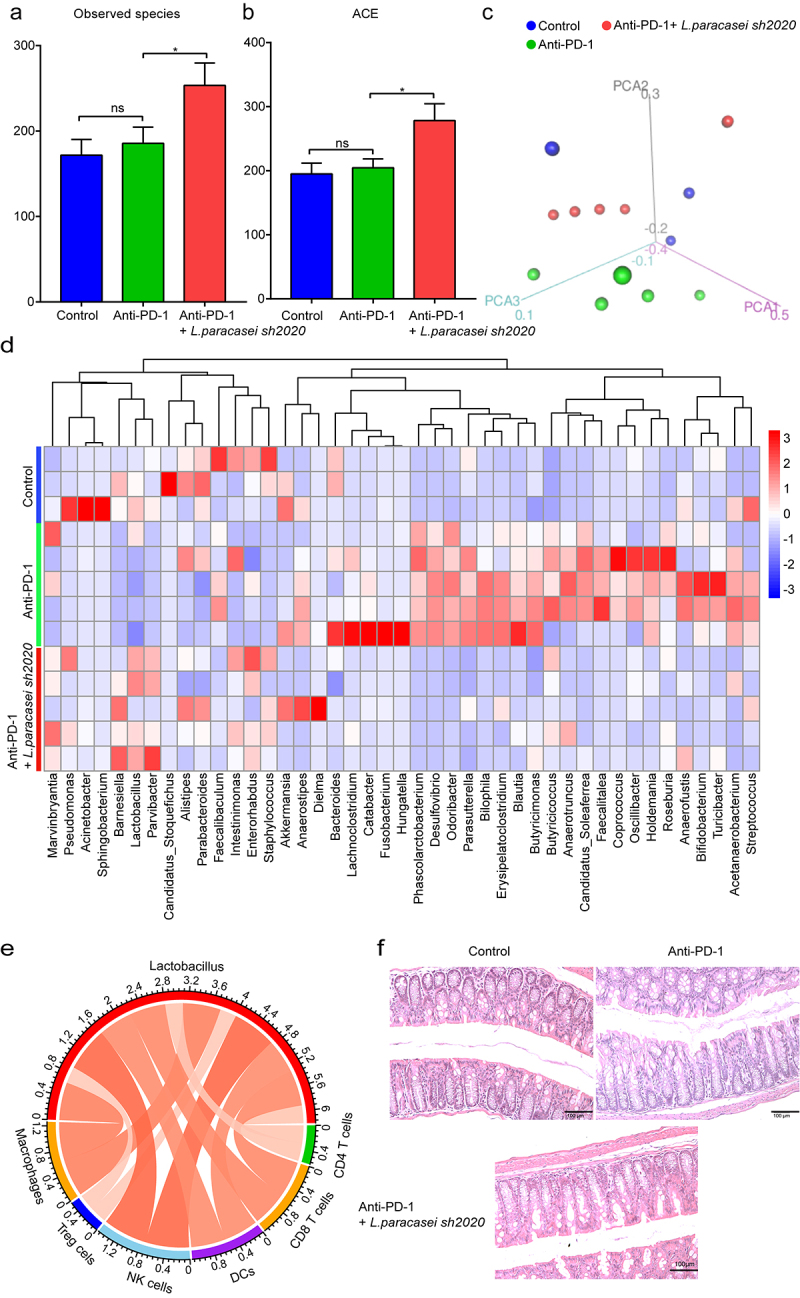
Oral administration of *L. paracasei sh2020* improved gut microbiota homeostasis in the context of anti-PD-1 therapy. (a-b) The α-diversity differences between the groups were estimated by the observed species (a), and ACE (b) (n = 3–5). (c) 3D-PCoA analysis of fecal samples based on the Bray-Curtis dissimilarity metric. (d) Community heatmap analysis at the genus level among different treatment groups (n = 3–5). (e) Correlation networks of *Lactobacillus* and tumor-infiltrating immune cells. (f) Representative images of H&E staining of the colon tissues in each group. ns, no significant difference, *P < .05.

ral intake of live probiotics can be expected to influence the gut homeostasis.^31^ Finally, we performed 16S rRNA sequencing to characterize the effects of *L. paracasei sh2020* oral administration on gut microbiota profiles. Oral intake of *L. paracasei sh2020* increased bacterial community diversity in the context of anti-PD-1 therapy (Figure 9a-b). 3D-PCoA analysis suggested that the overall gut bacterial community of the anti-PD-1 + *L. paracasei sh2020* group had gradually deviated from the control or anti-PD-1 group (Figure 9c). And the abundance of gut bacteria was considerably changed by *L. paracasei sh2020* in context of anti-PD-1 therapy (Figure 9c). *L. paracasei sh2020* increased the relative abundance of *Lactobacillus* in the anti-PD-1-treated mice. Furthermore, the correlations between *Lactobacillus* and the level of tumor-infiltration immune cells were also testified, confirming the strongly positive correlation between *Lactobacillus* and antitumor immune response (Figure 9e). In addition, H&E staining showed that *L. paracasei sh2020* might help to maintain gut homeostasis (Figure 9f), thereby providing an intact gut barrier against immune checkpoint inhibitor-associated diarrhea and colitis.^32^

## Discussion

Gut microbiota is heavily influenced by both genetic and environmental factors including age, gender, genotype, diet, and environmental exposures.^33^ It also emerged from several recent studies that different research institutions or commercial vendors usually harbor considerable variations in microbiological environment,^15,34^ leading to differences in gut microbiota structure and composition. Since the microbiome deficit of one individual may not necessarily mirror that of another individual, it is not surprising that patients respond differently to FMT. In this context, humanized microbiome mice model is a valuable donor-recipient matching approach for identifying the “super-donor”, whose stool confers significantly more successful FMT outcomes than the stool of other donors. In our study, we performed FMT using individual stool from five healthy donors, giving a personalized cocktail of organisms that were fed to the mice. One strength of our study could be that all human donors were young, and all of them have led a healthy lifestyle, including healthy balanced eating, regular physical activities, proper sleep, quit smoke and alcohol, and staying happy and positive. Studies have shown that a healthy functional microbial ecosystem is heavily shaped by these healthy lifestyles, which is fundamental to instruct the immune system toward homeostasis. Hence, it is of significance to screen the “super-donor” among this population, and thus guarantee FMT success in CRC patients underwent immunotherapy. Interestingly, even in the young and healthy individuals with healthy lifestyle, their gut microbiota differently influenced tumor progression in the presence of anti-PD-1 mb. The observed inter-individual variation among these donors was accompanied by specific gut microbiota profiles as well as T cell phenotypes in the TME. However, the precise mechanisms underlying these variations in recipient mice need to be investigated in future detail, focusing on individual microbiotas and specific pathways. Additionally, the number of healthy individuals in our study was small, and future studies with larger cohorts are required.

Just as a side water and soil raises side people, different environments and customs influence the gut microbiota structure and composition. Since we and other researchers have demonstrated that anti-PD-1 outcomes are heavily influenced by gut microbiota,^35,36^ it is not surprising that patients from different countries and regions respond differently to anti-PD-1 therapy. Moreover, the effector bacteria that are associated with good response to anti-PD-1 remain as high variation among different research institutions or commercial vendors. For example, *A.muciniphila* was reported to facilitate anti-PD-1 in metastatic melanoma patients.^37^ However, *A.muciniphila* abundance was not significantly changed after FMT from healthy donors in our study, indicating that the favorable antitumor effect of healthy gut microbiota did not follow the *A. muciniphila* way. In our study, we cultured and isolated a novel *L. paracasei* from the feces samples of healthy donors, which was named *L. paracasei sh2020*. We found that *L. paracasei sh2020* was effective to reduce tumor growth when combined with anti-PD-1. Surprisingly, single *L. paracasei sh2020* even produced a stronger antitumor effect than the anti-PD-1 therapy alone.

Tumor-infiltrating lymphocytes (TILs) represented the major component of the tumor microenvironment. Recent studies have divided solid tumors into two subtypes: “hot” tumor and “cold” tumor.^38,39^ The latter, with less TILs, usually represented poor response to immunotherapy,^40^ which was a major obstacle to effective treatment with anti-PD-1 among CRC patients.^41,42^ And researchers tried to develop strategies to turn “cold” tumors into “hot” tumors these days. Furthermore, improving T cell infiltration in the tumors via manipulating gut microbiota is an area of active research, which still stays at its infant stage. In our study, selective depletion of T cell subsets highlighted the indispensable role of CD8^+^ T cells in the *L. paracasei sh2020* treatment, which was also confirmed in the patients treated with anti-PD-1. Thus, we concluded that the effect of *L. paracasei sh2020* was largely dependent on CD8^+^ T cell in the tumors. Thus, increased T cell infiltration induced by *L. paracasei sh2020* was a promising strategy to improve poorly infiltrated tumor microenvironment, transforming “cold” tumors to “hot” ones.

Furthermore, we strived to clarify the precise mechanisms for responding to *L. paracasei sh2020*. We compared gene expression profiles in the *L. paracasei sh2020-*treated tumors with that in the control tumors, and GSEA of these genes highlighted Leukocyte migration-associated pathways as the key influenced pathway mediated by *L. paracasei sh2020*. Most noteworthy was that chemokines involved in these pathways also showed significance in a comparison of gene expression profiles between the control and *L. paracasei sh2020-*treated tumors, indicating that *L. paracasei sh2020* might promote T cell infiltration through reversing the expression of these genes. In fact, chemokines play crucial roles in the lymphocyte recruitment within the tumor microenvironment.^43^ More importantly, we identified that CXCL10 was the as the most significantly influenced chemokines by *L. paracasei sh2020*. CXCL10 was a T helper 1 type chemokine and governed the trafficking of the main antitumor immune cells, including CD8^+^ T cells, into the tumor beds. Considering this, we confirmed that *L. paracasei sh2020* treatment confers high levels of CXCL10 than the control tumor in both vitro and vivo. In the *in vitro* tumor cell culture model, stimulation by *L. paracasei sh2020* could result in the increased production of CXCL10, which was further confirmed by the *L. paracasei sh2020-*treated tumors *in vivo*. Hence, *L. paracasei sh2020* in antitumor immunity or as immunomodulator to facilitate immunotherapy.

In a review of the oral administration route for *L. paracasei sh2020*, we consequently outlined gut bacterial community in the mice using 16S rRNA gene sequencing. Oral administration of *L. paracasei sh2020* significantly altered the microbial community and increased the microbial community diversity in the context of anti-PD-1 therapy, which had favorable roles in the improved antitumor immunity. A healthy gut environment, shaped by the balanced gut microbiota, is fundamental to the presence of normal gut barrier.^20^ However, anti-PD-1 can cause immune-related adverse effects, especially in the patients with the dysbiosis of gut microbiota.^44^ One of the most common toxicities is anti-PD-1-associated colitis. Here, histopathological analysis of colons confirmed that *L. paracasei sh2020* administration might help to maintain intestinal homeostasis by modulating gut microbiota, which was a novel and promising strategy to mitigate the immune checkpoint inhibitor-associated colitis.^32^ In all, *L. paracasei sh2020* could ameliorate gut microbiota changes induced by anti-PD-1 therapy, and thus restore the homeostasis of gut microecology.

In conclusion, our study identified a novel commensal *L. paracasei sh2020* that was able to trigger anti-tumor immunity and prevent tumor growth in the mice. anti-PD-1 combined with *L. casei sh2020* markedly trig tumor suppression, making it superior to anti-PD-1 or *L. paracasei sh2020* monotherapy. Notably, the modulation of gut microbiota caused by *L. paracasei sh2020* might play a synergic role in enhancing immunotherapy.

## Materials and methods

### Cell line and culture

Mouse colon adenocarcinoma cell-line MC38 was obtained from Type Culture Collection of Chinese Academy of Sciences, China (Shanghai). The cell was cultured in the RPMI-1640 medium (Gibco, USA) with 10% fetal bovine serum (Gibco, USA) and 1% penicillin/streptomycin (Hyclone, USA) in a humidified atmosphere containing 5% CO2 at 37°C.

### Mice

All animal experiments were approved by the Ethics Committee of Minhang Hospital, Fudan University. The female C57BL/6 mice (6 to 8-weeks old) were obtained from Charles River Laboratories, China. All mice were housed under specific pathogen-free conditions and allowed to acclimate for one week before performing experiments. The ethics of animal research has been reviewed and approved by the Ethics Committee of Fudan-Minhang academic health system (Approval number: 2020 Minhang Hospital JS-006).

### Fecal microbiota transplantation (FMT) experiment

Five patients newly diagnosed with CRC and healthy volunteers were enrolled in the study, according to the criteria described previously.^34^ All Subjects information was provided in Table S1. Fresh stools from each subject were immediately suspended in an equal volume of PBS containing 20% glycerol, snap-frozen in liquid nitrogen, and stored in −80°C refrigerator until use. FMT was performed as follows: we gave antibiotics to adult mice (6–8 weeks of age) by oral gavage with 200 mg/kg of ampicillin, metronidazole, and neomycin, and 100 mg/kg of vancomycin daily for 3 days. Then, stool samples for humans were thawed and suspended in an equal volume of PBS, vortexed, and the supernatant was centrifuged. 100 µL of the fecal suspension was administered to mice by oral gavage, and another 100 µL was applied on the fur of each animal for 3 days. The study protocol has been reviewed and approved by the Ethics Committee of Fudan-Minhang academic health system and informed consent was obtained from all participants.

### Bacteria stains isolation, and identification

Fecal sample was homogenized in sterile physiological saline in a 15 ml sterile centrifuge tube. After centrifugation (1000 g, 1 min), the supernatant was incubated in a modified MRS medium for 18–24 h. After which, 10 µL of the enrichment cultures were streaked on the Rogosa agar, a highly selective media for the isolation of *Lactobacillus* spp,^45^ and incubated at 37°C for 24–48 h under aerobic conditions. After incubation, a sterile inoculation needle was used to pick up single colonies and streak them on clean plates to obtain pure cultures. Genome DNA was isolated for 16s rRNA PCR to verify the purified strain type. As for whole-genome sequencing, the bacterial genomic DNA was extracted for Oxford Nanopore and Illumina sequencing. Sequence blasts were carried out in the National Center for Biotechnology Information (NCBI) database. The high-quality reads were assembled by Miniasm.^24^ The genomes data for other *Lactobacillus* strains available in the NCBI database were downloaded for the comparative analysis.

### Mouse tumor models and treatments

When MC38 cells grew reaching about 80% confluence, they were harvested and washed three times in phosphate-buffered saline (PBS). 5.0 × 10^5^ cells were inoculated subcutaneously into the right flank of each mouse. Tumor volume and mouse weights were monitored every 3 days. Tumor volume was calculated as length × width^2^ × 0.5. The survival status of these mice was checked and recorded every day. For anti-PD-1 treatment, mice were intraperitoneally injected with anti-PD-1 (J43, BE0033-2, BioXcell) at a dose of 200 μg/mouse every 3 days for three times.

### The bacterial culture and administration

The isolated bacteria were resuspended and cultured in MRS broth at 37°C for 16–24 hours. Supernatants from the media were harvested, passed through a 0.45 µm filter, and either stored at −80°C or used directly for coculture with tumor cells. The strains were then centrifuged at 8000 g for 5 minutes and the pellets were diluted in the PBS. Each mouse was treated once-daily with 1.0 × 10^9^ CFU (*L. plantarum, L. paracasei*, and *L. reuteri*) cocktail for 15 days by oral gavage.

### Depletion of T cell subsets and CXCL10 in vivo

In the in vivo T cell subsets depletion study, InVivoMAb anti-mouse CD4 (YTS191, BE0119, BioXCell), CD8α (YTS169.4, BE0117 BioXcell) antibodies were intraperitoneally injected at a dose of 200 μg per mouse. To neutralize Cxcl10 *in vivo*, 50 µg of anti-mouse Cxcl10 (134013, MAB466, R&D systems) was intraperitoneally injected into mice every 3 days for 2 weeks.

### 16S rRNA sequencing analysis

Total bacterial DNA was extracted from fecal samples using the DNA extraction kit (TIANGEN, China). For analysis of the taxonomic composition of gut microbiota, the hypervariable regions V3-V4 region of 16S rRNA genes was pooled and sequenced at the Illumina MiSeq 2500 platform (Illumina MiSeq, USA). Reads were trimmed and classified using QIIME (V 1.8.0).^46^ After quality filtering and chimera removal, these sequences were assigned to operational taxonomic units (OTUs) with ≥ 97% similarity using UPARSE^47^ and RDP classifier^48^ was used to classify OTUs at a given taxonomic rank. The gut microbial signature was identified using *selbal*,^49^ a greedy stepwise algorithm for balance selection, and unsupervised RandomForest classification analysis.

### Next generation RNA sequencing (RNA-seq) analysis

Total RNA was extracted from tumors that were treated with or without *L. casei sh2020*. RNA-seq was performed using Illumina HiSeq (Novogene Bioinformatics Technology Co., Ltd.). After quality control, RNA-seq reads were mapped to the mouse reference haploid genome sequence (GRCm38.p3 C57BL/6, NCBI). Quantified transcripts were collapsed into gene counts, which were subsequently transformed to normalized FPKM values for all downstream analyses. Differentially expressed genes were identified using “limma” R package.

To explore signaling pathways enrichment, Gene Set Enrichment Analysis (GSEA) was performed using GSEA software version 4.0.0. The CIBERSORT algorithm^50^ was used to estimate the relative fractions of 25 distinct immune cell types within a complex mixture of RNA-seq data using deconvolution algorithm.

### Immunohistochemistry (IHC)

Formalin-fixed, paraffin-embedded tissues were sectioned at 5 μm thickness. And then the sections were dewaxed and repaired. The primary antibodies included rat anti-mouse CD8α (1:400, eBioscience,14–0808), rabbit anti-mouse CD4 (1:400, Abcam, ab183685), rabbit anti-Foxp3 (1:400, Abcam, ab54501), and rabbit anti-CXCL10 (1:800, absin, 135937). The staining intensity was scored from 0 to 3 (no staining, 0; weak staining, 1; moderate staining, 2; strong staining, 3). The staining extent was scored from 0 to 4 according to the positive proportion of cells (0%, 0; 1–25%, 1; 26–50%, 2; 51–75%, 3; >76%, 4). The final score was calculated as multiplying the staining intensity and extent score.

### Flow cytometry

Fresh tumor tissues were minced into small pieces and digested in an RPMI medium containing collagenase D (1 mg/ml; Roche) and DNase1 (150 UI/ml; Sigma) for 30 minutes at 37°C and then washed and filtered twice using 100 µm cell strainers (Falcon®). Fixable Viability Stain 620 (BD Biosciences) was used to discriminate live and dead cells. The cells were then blocked with Fc-block (BD Biosciences) and stained with anti-mouse antibodies. The following antibodies were used: PE/Cyanine7 anti-human/mouse CD45R (B220) (60–0452-U025, TONBO), Brilliant Violet 711^TM^ anti-mouse CD45 (103147, BioLegend), APC Anti-mouse NK1.1y (CD161) Antibody (20–5941-U025, TONBO), FITC Anti-mouse MHC Class II (I-A/I-E) Antibody (35–5321-U025, TONBO), PE Anti-mouse CD11c Antibody (N418) (50–0114-U025, TONBO), 710 Anti-mouse CD11b Antibody (M1/70) (80–0112-U025, TONBO), 450 Anti-mouse F4/80 (BM8.1) (75–4801-U025, TONBO), PE Anti-mouse/rat Foxp3 (FJK-16s) (12–5773-82, eBioscience), APC Anti-human/mouse/rat CD278 Antibody (ICOS) (APC ICOS 313510, Biolegend), eFluor 450 anti-mouse CD8*a* Antibody (48–0081-82, eBioscience), FITC anti-mouse CD4 Antibody (GK1.5) (11–0041-82, eBioscience), and APC anti-mouse INF gamma Antibody (XMG1.2) (17–7311-82, eBioscience).

### RNA extraction and quantitative real-time PCR (qRT-PCR)

Total mRNA was extracted using TRIzol reagent (Life Technologies). cDNA was synthesized by a PrimeScriptTM RT kit (Takara). RNA concentration purity was measured on a Nanodrop1000 spectrophotometer (Agilent). Quantitative real-time PCR was performed on cDNA using gene-specific primers in the presence of SYBR Green (Applied BioSystems). The results were analyzed the 2^−ΔΔCT^ method. Primer pairs used in this study were shown as follows:

**Table:** 

Genes	Forward (5’ – 3’)	Reverse (5’ – 3’)
CXCL9	GAAGTCCGCTGTTCTTTTCC	TTGACTTCCGTTCTTCAGTGT
CXCL10	GCTGCAACTGCATCCATATC	AGGAGCCCTTTTAGACCTTT ATGTAC
CXCL11	CTTATGTTCAAACAGGGGCG	TGCATTATGAGGCGAGCTT
*β-actin*	CGCAAAGACCTGTATGCCAAT	GGGCTGTGATCTCCTTCTGC

### Enzyme linked immunosorbent assay (ELISA)

For the quantitative determination of CXCL10 secreted protein levels in the tumor cell supernatants, as well as in the serum of mice, we used the Mouse CXCL10/IP-10 ELISA Kit (MULTISCIENCES, EK268/2), according to the manufacturer’s instructions.

### Statistical analysis

All data were presented as the mean ± SEM. Differences between groups were determined by two-tailed unpaired Student’s t-test using GraphPad Prism 7.0 software or as otherwise stated in the figure legend.

## Supplementary Material

Supplemental Material

## Data Availability

All data relevant to the study were included in the article or uploaded as supplementary information.
