# Supplementary figures and images for: Lacticaseibacillus paracasei sh2020 induced antitumor immunity and synergized with anti-programmed cell death 1 to reduce tumor burden in mice
Source: Gut Microbes. 2022 Mar 8;14(1):2046246. doi: 10.1080/19490976.2022.2046246 (PMC8920197; doi:10.1080/19490976.2022.2046246)

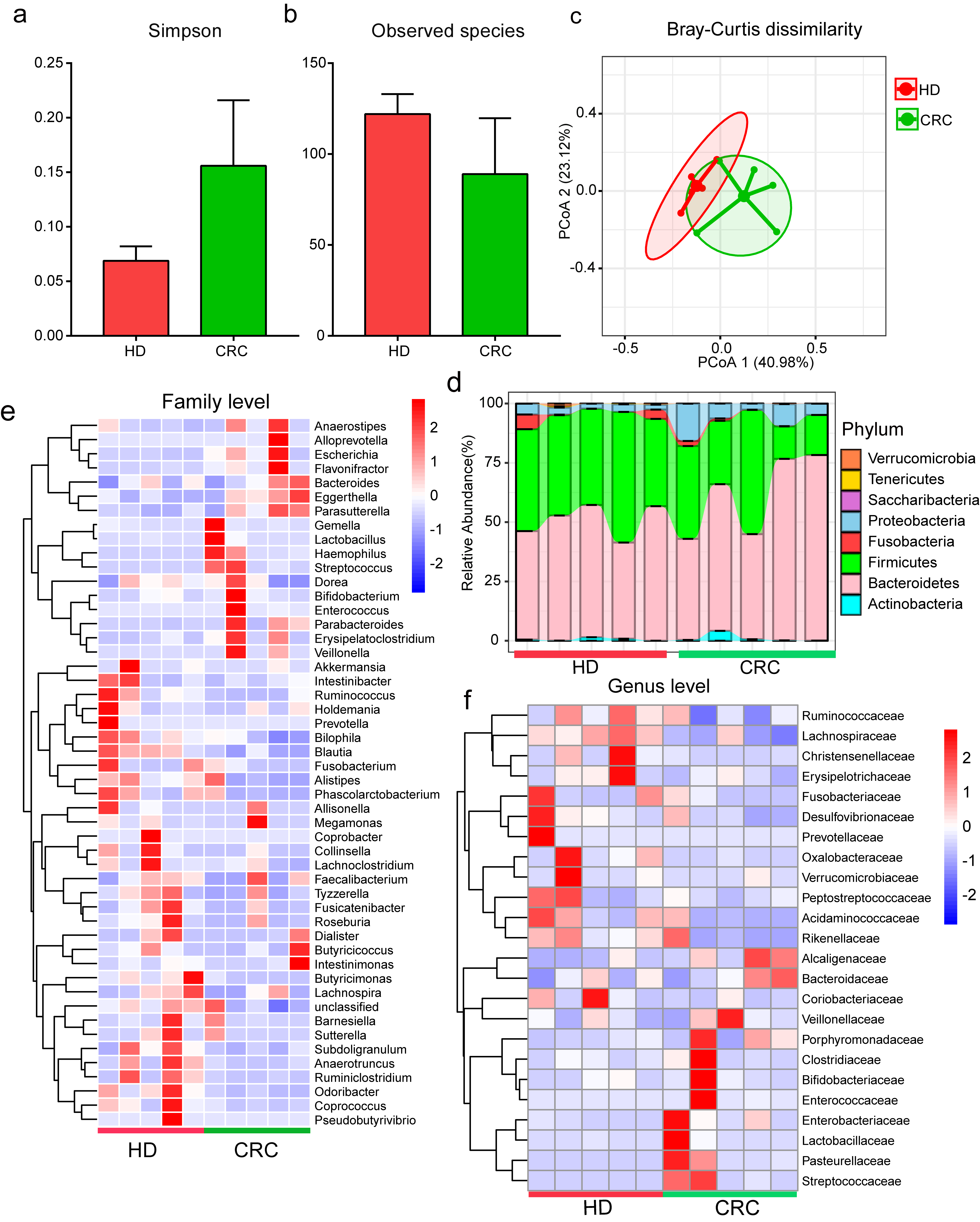

Supplement: Supplemental Material [file KGMI_A_2046246_SM4125.zip › Figure S1.tif]

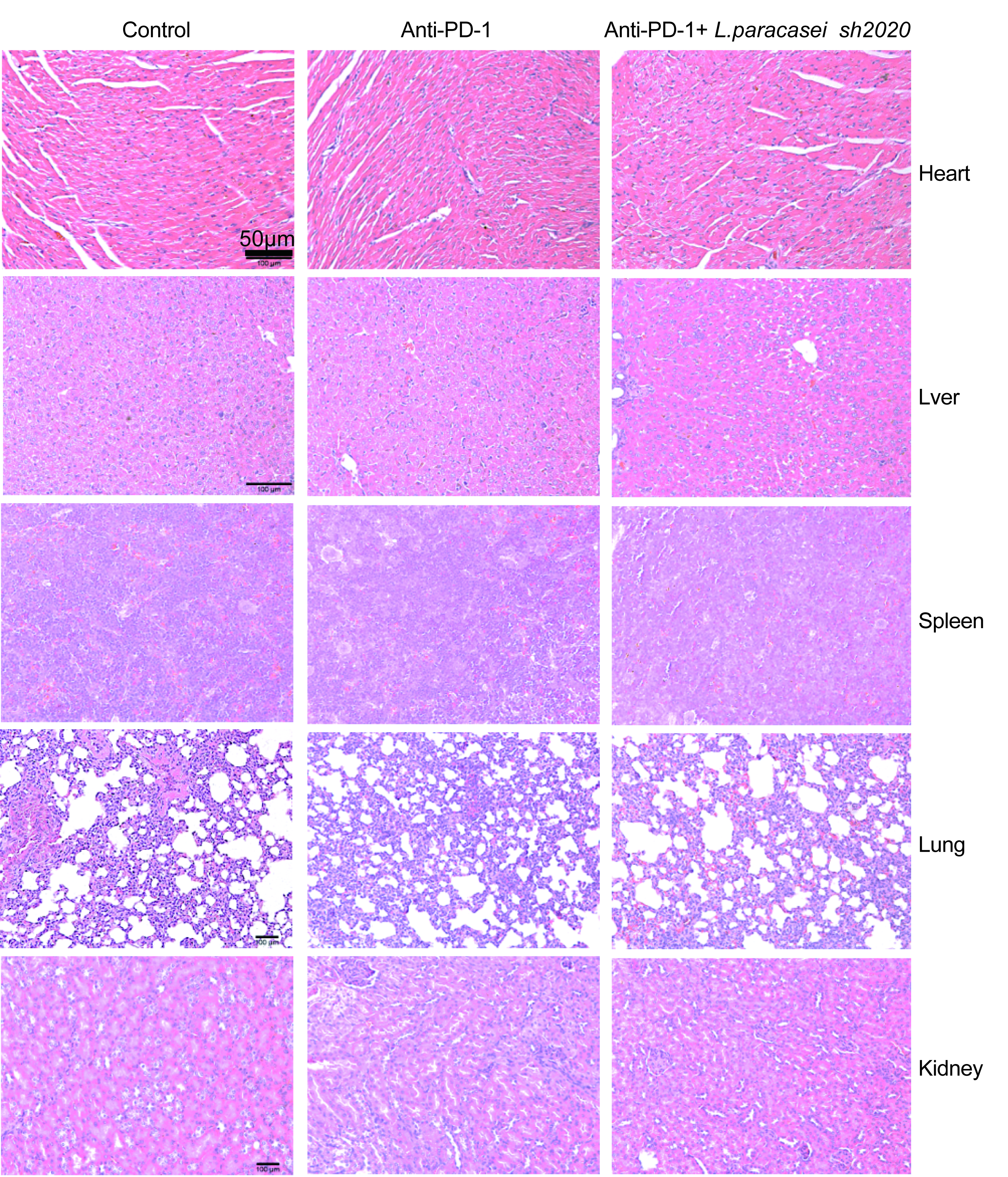

Supplement: Supplemental Material [file KGMI_A_2046246_SM4125.zip › Figure S2.tif]

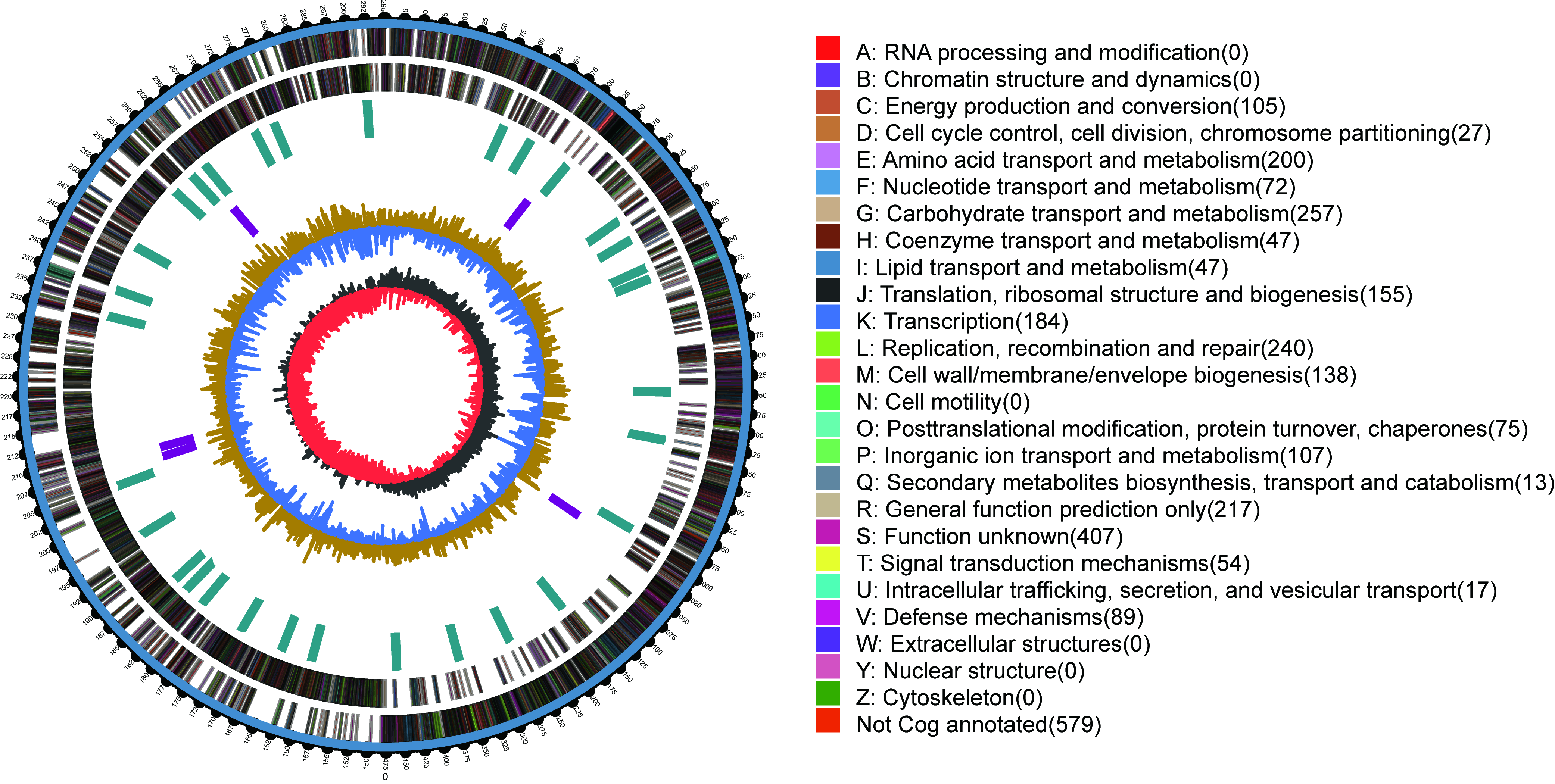

Supplement: Supplemental Material [file KGMI_A_2046246_SM4125.zip › FigureS3.tif]

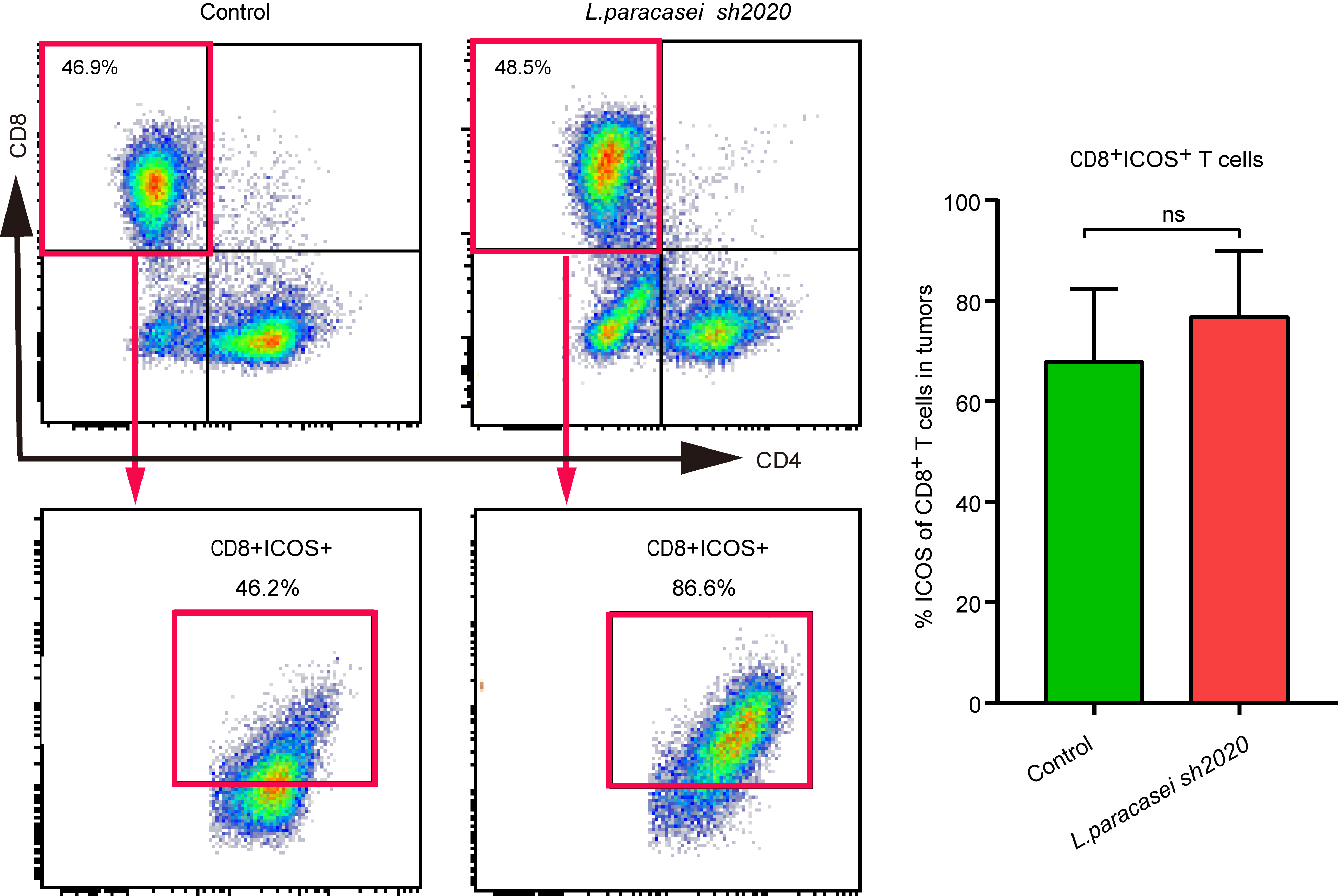

Supplement: Supplemental Material [file KGMI_A_2046246_SM4125.zip › FigureS4.tif]

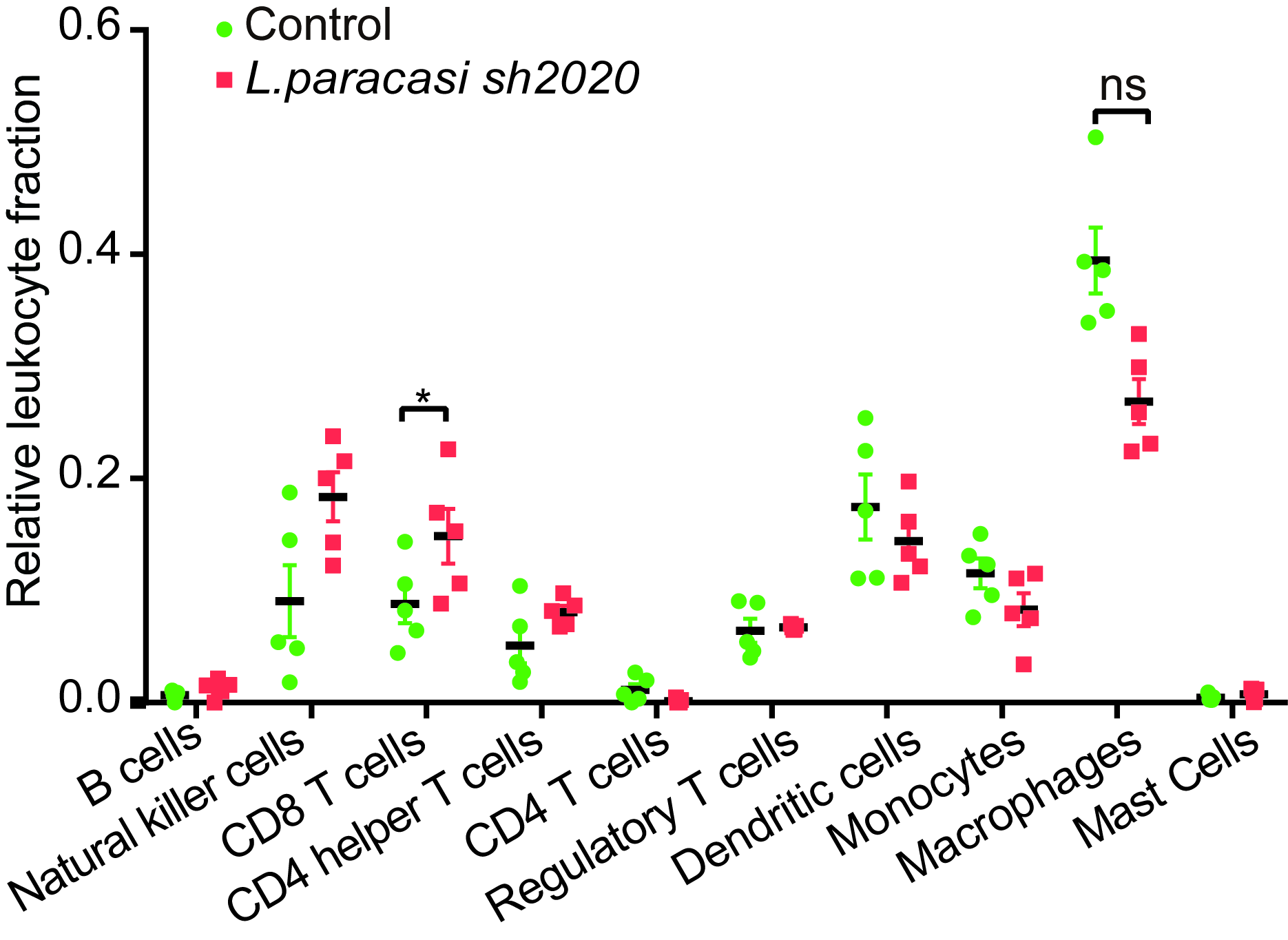

Supplement: Supplemental Material [file KGMI_A_2046246_SM4125.zip › FigureS5.tif]

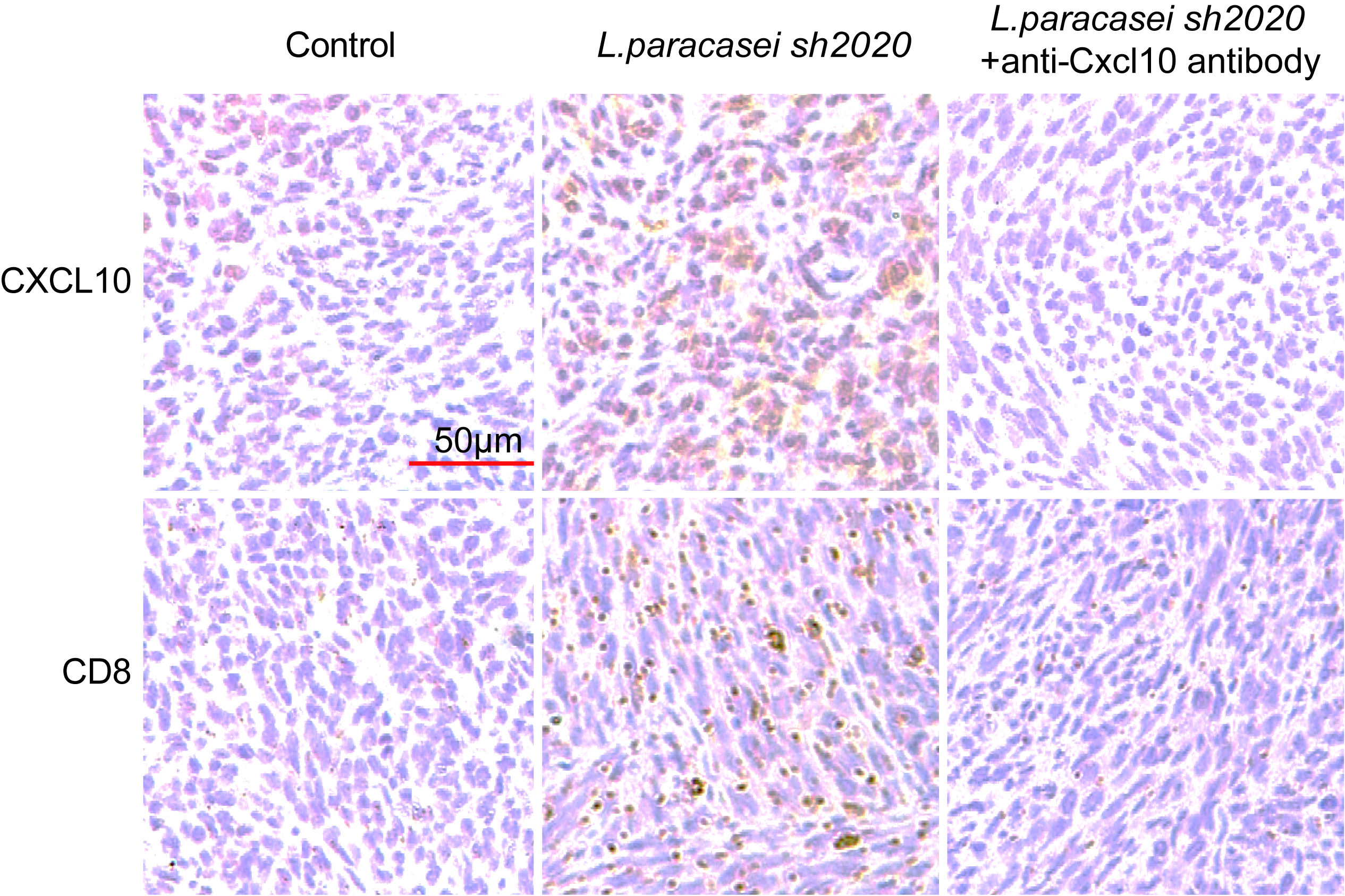

Supplement: Supplemental Material [file KGMI_A_2046246_SM4125.zip › FigureS6.tif]
